# Spectral characterization of wheat functional trait responses to Hessian fly: Mechanisms for trait-based resistance

**DOI:** 10.1371/journal.pone.0219431

**Published:** 2019-08-22

**Authors:** Veronica A. Campos-Medina, Lorenzo Cotrozzi, Jeffrey J. Stuart, John J. Couture

**Affiliations:** 1 Department of Entomology, Purdue University, West Lafayette, IN, United States of America; 2 Department of Forestry and Natural Resources, Purdue University, West Lafayette, IN, United States of America; 3 Center for Plant Biology, Purdue University, West Lafayette, IN, United States of America; Natural Resources Canada, CANADA

## Abstract

Insect herbivores can manipulate host plants to inhibit defenses. Insects that induce plant galls are excellent examples of these interactions. The Hessian fly (HF, *Mayetiola destructor*) is a destructive pest of wheat (*Triticum spp*.) that occurs in nearly all wheat producing globally. Under compatible interactions (i.e., successful HF establishment), HF larvae alter host tissue physiology and morphology for their benefit, manifesting as the development of plant nutritive tissue that feeds the larva and ceases plant cell division and elongation. Under incompatible interactions (i.e., unsuccessful HF establishment), plants respond to larval feeding by killing the larva, permitting normal plant development. We used reflectance spectroscopy to characterize whole-plant functional trait responses during both compatible and incompatible interactions and related these findings with morphological and gene expression observations from earlier studies. Spectral models successfully characterized wheat foliar traits, with mean goodness of fit statistics of 0.84, 0.85, 0.94, and 0.69 and percent root mean square errors of 22, 10, 6, and 20%, respectively, for nitrogen and carbon concentrations, leaf mass per area, and total phenolic content. We found that larvae capable of generating compatible interactions successfully manipulated host plant chemical and morphological composition to create a more hospitable environment. Incompatible interactions resulted in lower host plant nutritional quality, thicker leaves, and higher phenolic levels. Spectral measurements successfully characterized wheat responses to compatible and incompatible interactions, providing an excellent example of the utility of Spectral phenotyping in quantifying responses of specific plant functional traits associated with insect resistance.

## Introduction

Insect herbivores often manipulate plant physiology to minimize resistance and successfully colonize a host. Plants recognize herbivory through a number of mechanisms and can initiate inducible defense responses to limit herbivory while herbivores can utilize mechanisms to limit plant defense responses and increase nutritional quality [1] Herbivore-induced changes in plant functional traits, including nutritional and defensive composition, can have either positive or negative effects on herbivore performance.

Insect-induced plant galls represent a prime example of plant-herbivore interactions whereby the herbivore modifies plant physiology to successfully utilize the plant as a host [2]. Insect-induced galls are often considered an extended phenotype of the herbivore, as gall formation is greatly influenced by insect gene products [3]. Galling insects alter plant tissue formation to construct the gall, but changes in plant chemical composition, including nutritional, volatile, and other non-volatile secondary metabolites, also occurs with potential influence for insect performance and susceptibility to natural enemies [2, 4–7].

The Hessian fly (HF, *Mayetiola destructor*) is a gall-inducing insect and a destructive pest of wheat (*Triticum spp*.) [8]. Since its introduction into the United States, this pest has spread to all wheat producing areas of the country [9]. Its economic significance has encouraged its study and this in turn has made the HF a good model for investigations of the factors that insects use to induce plant galls. The genetic tractability of the insect revealed that effector-triggered immunity (ETI) to the insect exists in the plant [10, 11]. Wheat contains resistance (*R*) genes whose products detect specific effector proteins encoded by corresponding *Avirulence (Avr)* genes in HF. These effectors are secreted into plant cells as the insect larvae attempt to feed. When *R* gene products detect their cognate effectors (the incompatible interaction), they elicit a highly effective resistance response that kills the attacking larvae. However, when either the plant *R* gene or the insect cognate *Avr* gene are lacking (i.e., a compatible interaction) the plant is galled and the larvae survive.

To date, the *H13 R* gene and the *vH13 Avr* gene constitute the best studied cognate gene combinations between wheat and HF. *H13* has been physically positioned within a cluster of nucleotide-binding site leucine-rich repeat (NBS-LLR) resistance genes on chromosome 6DS in wheat [12]. Near isogenic wheat lines that either lack *H13* (Newton) or carry *H13* (Molly) have been developed [13] that facilitate the study of both *H13*-compatible and -incompatible interactions. *vH13* has also been genetically and physically positioned in the HF genome [14]. *H13*-avirulent flies carry the dominant “H13-avirulence” allele (*vH13*^*v*^) at the *vH13 Avr* locus. These alleles produce the vH13 effector protein, which is presumably detected by the product encoded by the wheat *H13 R* gene [10]. Distinct HF genotypes that either lack (Great Plains, or GP) or carry (vH13-US and vH13-ISR) the functional *vH13* allele have been isolated from both US (GP and vH13-US) and Israeli (vH13-ISR) populations.

Past investigations have characterized both *H13*-compatible and -incompatible interactions using microscopy [15–17] and gene expression analyses [18]. Under compatible interactions, *H13*-virulent larvae induce changes that affect the plant phenotype. Galled plants exhibit stunted growth and changes in leaf color, likely an outcome of changes in plant nutritional quality at the beginning of the infestation. The induction of nutritive tissue galled tissue is believed to act as a sink for nutrients that are mobilized through the vascular system, supplying high quality resources to the larvae with a detrimental effect on the plant development [15, 19]. Plant cell permeability is clearly increased throughout the vascular system in response compatible interactions [16]. On the other hand, under incompatible interactions *H13* prevents the induction of localized nutritive tissue and the larvae are not able to establish [16], and plants do not express changes consistent with compatible interactions [20]. Gene expression analyses indicate that other changes in plant metabolism occur [18]. These include alterations in carbon and nitrogen metabolism, however these changes are not associated with production of volatile compounds [5]. Phenolic compounds have been associated with feeding deterrence by cereal aphids in winter wheat [21], but it is unclear whether these play a role in HF resistance. The phenotypic responses elicited by HF to create a more favorable environment to enhance growth and development, however, are poorly characterized [6, 7] and represent a knowledge gap in developing mechanistic underpinnings of wheat resistance.

Reflectance spectroscopy is emerging as an effective method to estimate plant responses to stress [22, 23] including responses to the environment, insects, and pathogens [24–27]. The approach can also be used to determine changes in specific plant functional traits, including morphological and physiological responses and nutritional and secondary metabolite composition [24, 27–32]. This methodology is relatively less expensive and time consuming than standard analytical measurements and importantly, is non-destructive, allowing for repeated measurements.

The capacity of reflectance spectroscopy to identify and quantify plant chemical and physiological status is based on differential light absorption features across different chemical structures and functional groups. Absorption, and ultimately reflection, intensity of light from plant tissue across the visible (VIS, 400–700 nm), near-infrared (NIR, 700–1300 nm), and short-wave infrared (SWIR, 1300–2500 nm) depends on the changes in electron transition states, for VIS region, and differential vibrational overtones of molecular bonds, for the NIR-SWIR regions, specifically C-H, N-H, and O-H bonds, the main bonds associated with organic compounds [33]. Light that matches the frequency of these vibrational waves is absorbed, while light that does not match these frequencies is reflected or transmitted [29]. Knowledge of these interactions generates an understanding of plant chemical composition. Moreover, changes in plant chemical and physiological profiles in response to stress can be non-destructively quantified using this approach. Thus, this technology is emerging as a valuable alternative to standard analytical approaches to describe plant responses to stress.

While reflectance spectroscopy has been utilized to characterize induced plant defenses in response to damage in real time, to our knowledge, the approach has yet to be used to investigate the development of insect-induced plant galls. Here, we use this approach to investigate this process between wheat and HF, perhaps the best studied insect-plant galling interaction. We determine whole-plant changes in wheat functional trait profiles in response to HF infestation under both compatible and incompatible interactions, examine whether plant responses are consistent between different populations of virulent HFs and evaluate the ability of Spectral data to characterize these responses.

Here, we examine the impact of HF on wheat foliar nutritional and defensive characteristics to determine if changes in these functional traits are consistent with wheat resistance. We determine whole-plant changes in wheat functional trait profiles in response to HF infestation under both compatible and incompatible interactions, over a time course important for HF establishment, and examine whether plant responses are consistent between different genotypes of virulent HFs.

## Materials and methods

### Plant and insect material

Seedlings of the wheat line ‘Molly’ were used in all experiments. Molly is an *H13*-line that is nearly isogenic to the HF-susceptible line ‘Newton.’ Seedlings were germinated in autoclaved soil under standardized greenhouse conditions in 4-cm diameter × 14-cm depth plastics cones.

Two *H13*-virulent HF strains, or genotypes, were used. One virulent strain was selected from a United States population, hereafter referred to as vH13-US. The other virulent strain was selected from an Israeli population, hereafter referred as vH13-ISR. Additionally, we used an *H13*-avirulent genotype, Great Plains (GP) that is unable to survive on wheat plants with any known resistance genes.

### HF infestation protocol

Gravid females from the three strains were allowed to lay their eggs on Molly seedlings. Three days after oviposition, neonate first-instar larvae were collected in 10 μL of an aqueous solution containing 0.02% NP-40 and transferred to Molly plants at the two-leaf developmental stage (10–12 day old plants) using a micropipette. Each plant was infested with two larvae. To examine compatible interactions, 60 plants were infested with vH13-US and 60 plants with vH13-ISR HFs. To examine incompatible interactions, 60 plants were infested with GP flies. Forty uninfested plants served as a control. Plants were well-irrigated and maintained in a growth chamber at 20±2°C, 80% relative humidity, and a 14:10 light:dark photoperiod.

### Determination of wheat traits and collection of foliar tissue

We collected leaf reflectance using a high resolution full-range (350–2500 nm) spectroradiometer (1024i; Spectral Vista Corporation, Poughkeepsie, NY USA) using a reflectance probe with a leaf clip attachment. Because the area of an individual wheat leaf blade does not fill the leaf clip field-of-view, we used a destructive sampling method in which all of the leaf blades of individual plants were aligned side-by-side to form a leaf mat with no overlap, to create an area that filled the leaf clip field of view following Serbin et al. [34]. Two reflectance measurements were collected on the foliar adaxial surface for each grouping of leaves. We collected leaf reflectance data on plants immediately before infestation, and then measured reflectance from uninfested control plants, and infested plants in both compatible (virulent HFs) and incompatible interactions (avirulent GP HFs). Measurements were taken 4 and 12 days post infestation (dpi), when the plants were 14–16 and 22–24 days old, respectively and the larvae were in the first and third instar, respectively. The total number of plants examined was as follows: 45 pre-infestation plants, 40 uninfested control plants each at 4 dpi and 12 dpi, 45 plants from each virulent HF population at 4 dpi, 60 plants from each virulent HF population at 12 dpi, and 40 plants from avirulent HF genotype at 4 and 12 dpi. Immediately following spectral measurements, foliar samples were flash-frozen in liquid nitrogen and stored at -20°C and then lyophilized. After drying, leaf area was quantified using the program WhinRHIZO by scanning individual leaves on a calibrated scanner. The leaves were then weighed and leaf mass per area (LMA, mg cm^-2^) was calculated. The samples were then ground to a homogenous particle size using a ball mill.

### Chemometric modeling approach

We generated models to predict foliar carbon (C, % dry weight, DW), nitrogen (N, % DW), leaf mass per area (LMA, g DW m^-2^) and phenolic concentration (mg g^-1^ DW) using fresh leaf spectral data and partial least squares regression (PLSR; [35, 36]). In cases where predictor variables are highly correlated, such as with spectral data, traditional regression techniques produce unreliable coefficients, as predictor variables lead to bias in coefficients and error estimates [37]. PLSR, in contrast with standard regression techniques, reduces a large number of collinear predictor variables into a relatively low uncorrelated number of latent variables, and has become the preferred method for chemometric analyses [30, 34, 38–40]. We based the number of latent variables on the reduction of the predicted residual sum of squares (PRESS) statistic [41] using leave-one-out cross validation. We combined the final set of extracted factors into a linear model predicting chemical concentrations.

While we collected spectra from each plant, the limited amount of foliar tissue available for each plant forced us to subset, and in cases combine, samples in order to obtain enough tissue for specific chemical analysis (described below). Combined samples were always from the same treatment combination (i.e., same fly genotype-dpi combination) and if samples were combined for chemical analyses, the corresponding spectral data were averaged for use in chemometric modelling. Total number of samples for C, N, and total phenolics chemical analyses was 79; total number of samples for LMA determination was 343. Prediction residuals was used to identify potential outliers, following Couture et al. [32]. Spectral data of outliers were further examined for errors, which included elevated reflectance in the VIS region, misaligned detector splicing producing jumps, or concave spectral shape at the red-edge peak, all a result of the leaf clip not being fully closed during the reference or target measurement. Samples that were determined to be outliers were subsequently removed from further analyses (C = 5, 6% of dataset; N = 5, 6% of dataset; LMA = 33, 8% of dataset; phenolics = 4, 5% of dataset). Summary statistics for all functional traits can be found in S1 Table.

We assessed model performance by conducting 500 randomized permutations of the dataset using 70% of the data for internal calibration and withholding the remaining 30% for cross validation. For each model permutation, we tracked the model fit (*R*^*2*^), root mean square error (RMSE), and bias to assess model performance as applied to the held-out dataset. Here, we report the RMSE as both the determined value and as a percentage of the range of data (%RMSE) for each dependent variable. The latter metric is useful to assess the predictability error of the model within the data range used in the model and is also comparable across different models. In addition, we determined the strength of the contribution of PLSR coefficients by individual wavelengths using the variable important to the projection (VIP) statistic [35, 36]. The VIP indicates the importance of an individual wavelength in explaining the variation in the response and predictor and response variables, such that larger weightings confer greater influence of individual wavelengths to the predictive model [36, 42]. The modeling approach and model performance analyses were performed using the *pls* package [43] in R (www.r-project.org).

### Chemical analyses of foliar tissue

Standard analytical determination of foliar carbon and nitrogen was performed using a Thermo Finnigan Flash 1112 elemental analyzer (San Jose, CA, USA). Total phenolic contents were quantified colorimetrically according to Ainsworth and Gillespie [44] with minor modifications. Twenty mg of dried leaf samples were extracted with 1.9 ml of 95% (vol/vol) methanol at room temperature for 48 h in the dark. Extracts were centrifuged for 5 min at 13,000*g* and room temperature. Then, 100 μl of each sample supernatant were mixed with 200 μl of 10% (vol/vol) Folin-Ciocalteu reagent and 800 μl of 700 mM Na_2_CO_3_. After a 2-h incubation at room temperature, 200 μl of each sample were transferred in a clear 96-well microplate and absorbance of each sample was recorded at 765 nm using a microplate reader (SpectraMax 190, Molecular Devices, Sunnyvale, CA). The blank-corrected absorbances were quantified using a gallic acid standard curve (0–0.7 mg/ml) and are reported as gallic acid equivalents.

### Statistical analyses

We analyzed leaf N concentration, C:N ratios, LMA, and phenolic content by two-way analysis of variance (ANOVA) following the model *y*_*ij*_ = μ *+ I*_*i*_ *+ T*_*j*_ *+ IT*_*ij*_ *+ e*_*ij*_. In this model, μ represents the mean, *I* represents infestation status *i*, *T* represents time from infection *j*, *IT* represents the interaction between infestation status *i* and time from infestation *j*, and *e*_*ij*_ represents the error term. Treatments for this analysis included an uninfested control, both compatible genotypes (vH13-US and vH13-ISR) separately, and the incompatible population (GP), but did not include the pre-infestation measurements. To examine the changes in plant functional traits over time, relative to the average pre-infestation value, we used a similar two-way ANOVA approach as described above with the exception that we used percent change in plant responses to the pre-infestation measurements. In this analysis, compatible HF populations were averaged and used as a single treatment. Pre-infestation values were averaged and then percent change of individual plants was calculated as ((mean pre-infestation value–treatment value)/mean pre-infestation value)*100. Treatments for this analysis included an uninfested control, both combined compatible populations (vH13-US and vH13-ISR), and the incompatible population (GP). We additionally compared the influence of the different compatible HF genotypes on plant responses using a separate, but similar, two-way ANOVA analysis as above with the exception that the treatment contrasted only relative changes between the two compatible HF genotypes and the average pre-infestation value. If the ANOVA was significant, we examined differences between treatments using Tukey *post-hoc* tests. Treatments for this analysis included individuals from the vH13-US and vH13-ISR genotypes. Statistical analyses were performed in JMP v13.2 (SAS Institute Inc., 2016).

## Results

### Chemometric modelling

**Fig 1:**
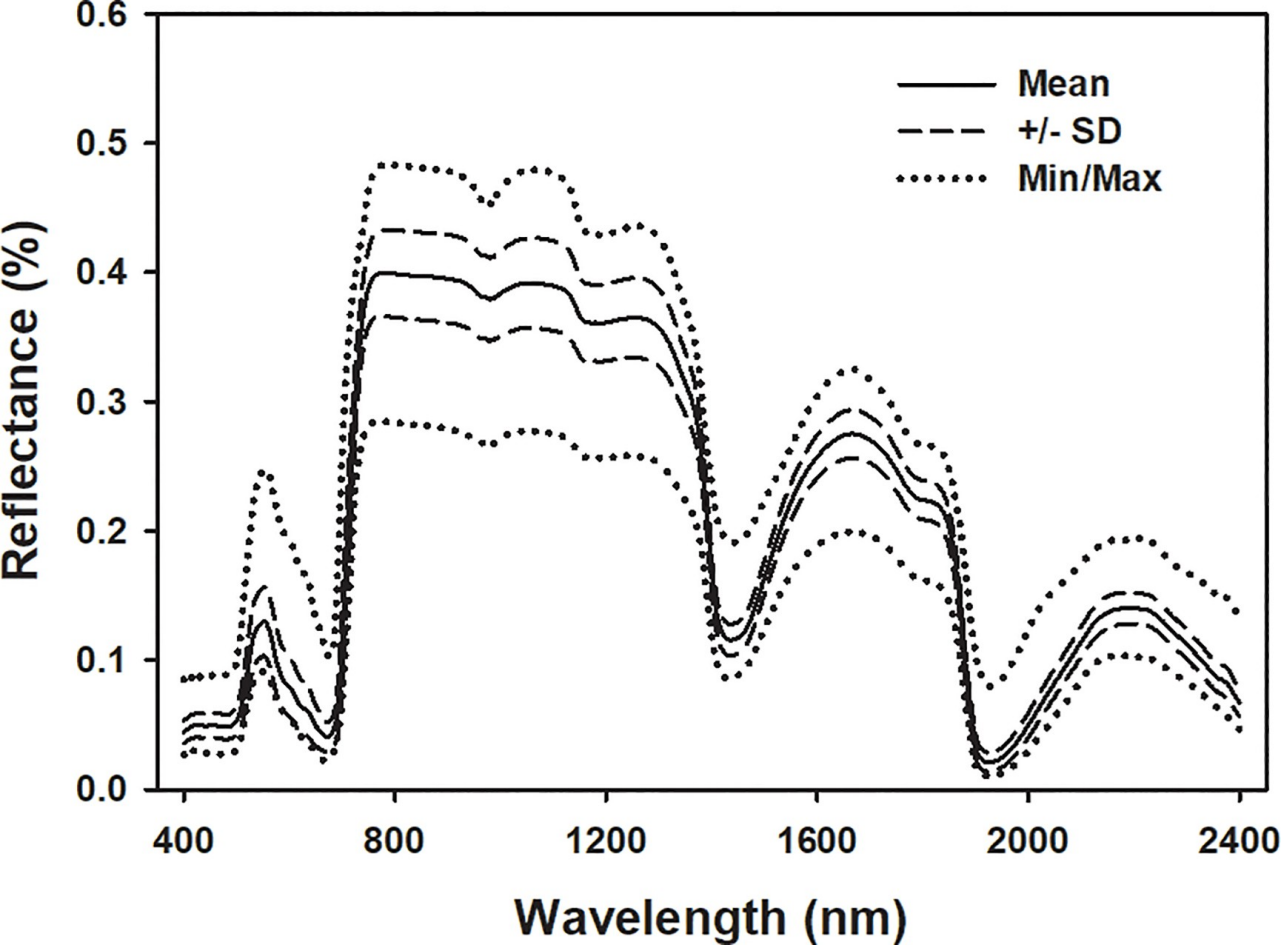
Mean, ± standard deviation (SD), minimum (Min), and maximum (Max) wheat reflectance values.

**Fig 2:**
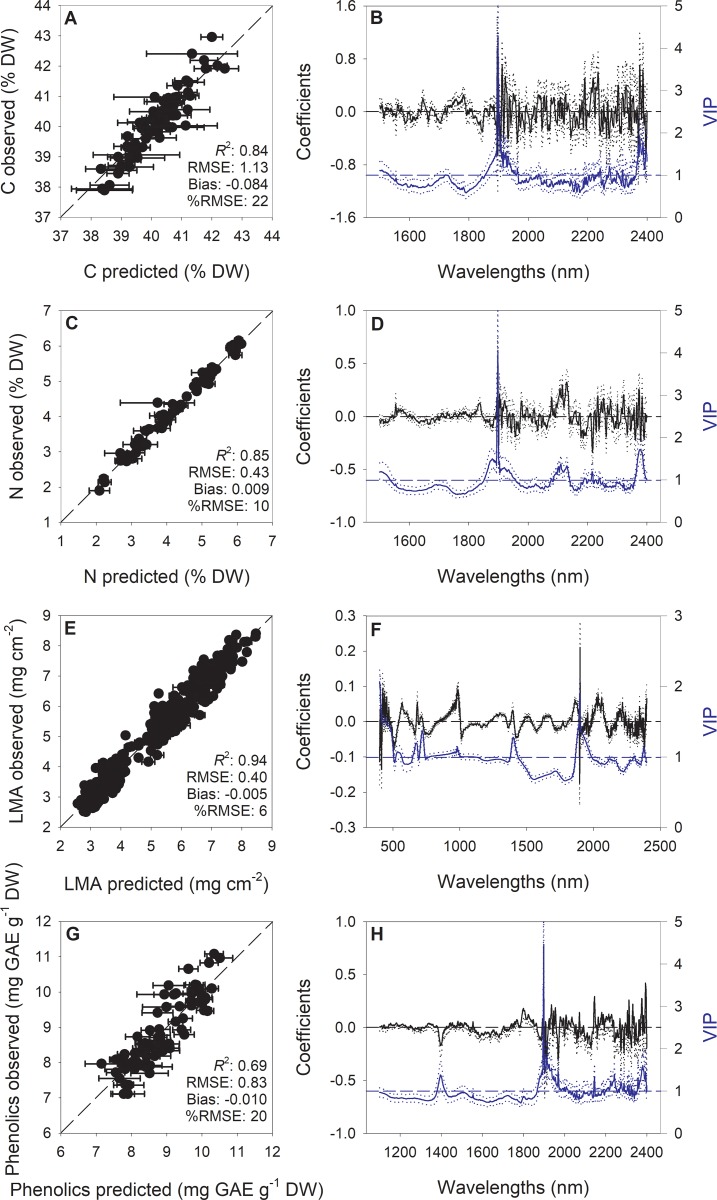
Observed vs predicted values, generated using partial least squares regression (PLSR), of foliar carbon (C), nitrogen (N), leaf mass per area (LMA) and phenolic concentrations in wheat (A, C, E, G); error bars for predicted values represent the standard deviations generated from 500 simulated models; dashed line is 1:1 relationship; mean model goodness-fit (*R*^*2*^), mean root-mean-square error (RMSE), mean bias, and mean %RMSE for cross validation data generated using 80% of the data for calibration and 20% for cross validation over the 500 simulations are reported. Mean (solid), 5^th^ and 95^th^ percentile (dotted) of standardized coefficients (black) and variable importance for projection values (VIP, blue) by wavelengths for PLSR-models predicting C, N, LMA and phenolics in wheat (B, D, F, H). GAE, gallic acid equivalents.

Substantial variation existed in wheat reflectance profiles across genotypes and treatments (Fig 1). We initially examined numerous models containing multiple wavelength ranges and components to optimize model performance. We finally used wavelengths between 1500 and 2400 nm to build models for N and C (11 and 13 components, respectively), 400–2400 nm for LMA (13 components), and 1100–2400 nm for phenolics (12 components). Carbon, N, LMA, and phenolics were well predicted from spectral data collected from leaves of wheat under different infestation treatments (uninfested, compatible, and incompatible; Fig 2). Mean cross validation values for C are as follows: *R*^*2*^, 0.84 (range: 0.74–0.98); RMSE, 1.13 (range: 0.66–1.80); bias, -0.084 (range: -0.75–0.85); %RMSE, 22. Mean cross validation values for N are as follows: *R*^*2*^, 0.85 (range: 0.43–0.96); RMSE, 0.43 (range: 0.24–0.96); bias, 0.009 (range: -0.27–0.34); %RMSE, 10. Mean cross validation values for LMA are as follows: *R*^*2*^, 0.94 (range: 0.90–0.96); RMSE, 0.40 (range: 0.32–0.47); bias, -0.005 (range: -0.13–0.11); %RMSE, 6. Mean cross validation values for phenolics are as follows: *R*^*2*^, 0.69 (range: 0.59–0.76); RMSE, 0.83 (range: 0.58–1.52); bias, -0.01 (range: -0.72–0.68); %RMSE, 20. The distribution ranges for *R*^*2*^, RMSE and bias also suggest that the spectroscopic models predicting all plant traits were relatively stable. Coefficient and VIP profiles highlighted important wavelengths around 1900 nm for all predicted traits, and also from 400 to 500 nm for LMA.

### Functional trait responses

**Fig 3:**
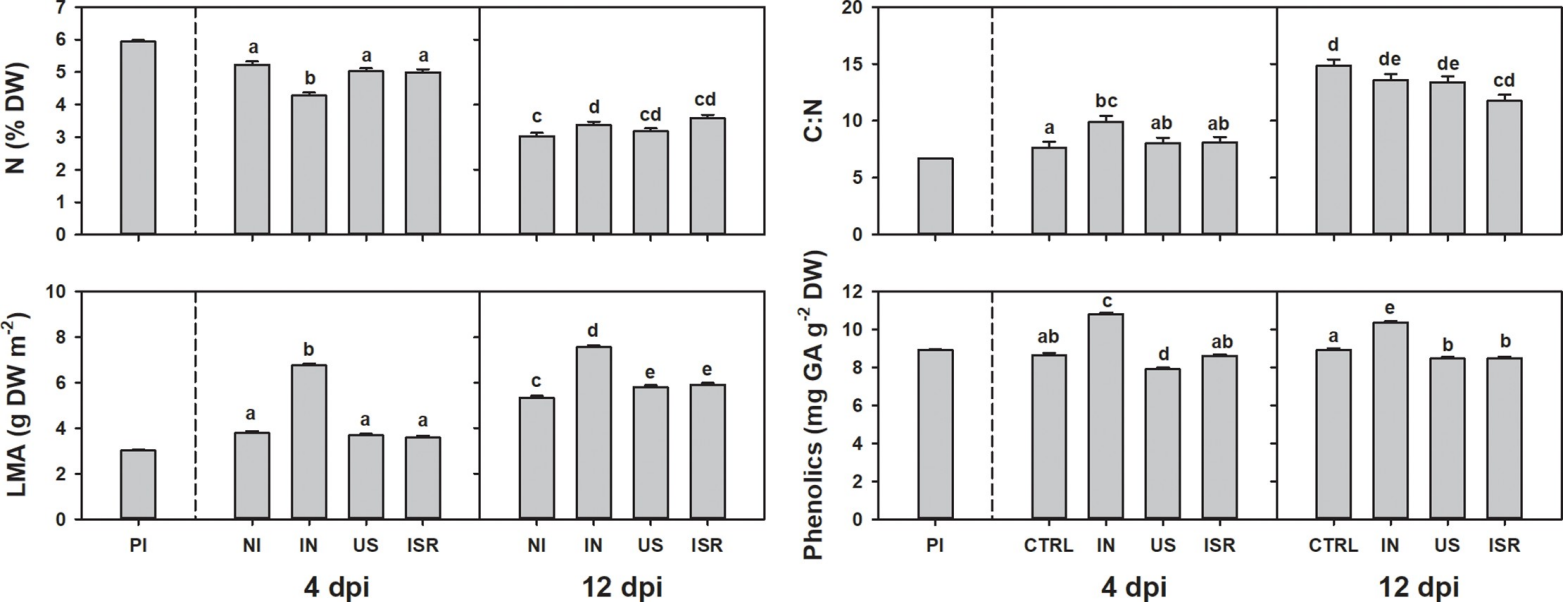
Mean ± SD of nitrogen (N), ratio of carbon to nitrogen (C:N), leaf mass per area (LMA) and phenolics at pre infestation (PI) and at four and twelve days post infestation (dpi) for the non-infested (NI) control, and plants with incompatible (IN) and compatible (H13-US, US; H13-ISR, ISR) populations. Statistical analysis includes only NI, IN, US, ISR. DW, dry weight. Treatments not connected by the same letter are significantly different (*p* > 0.05).

**Fig 4:**
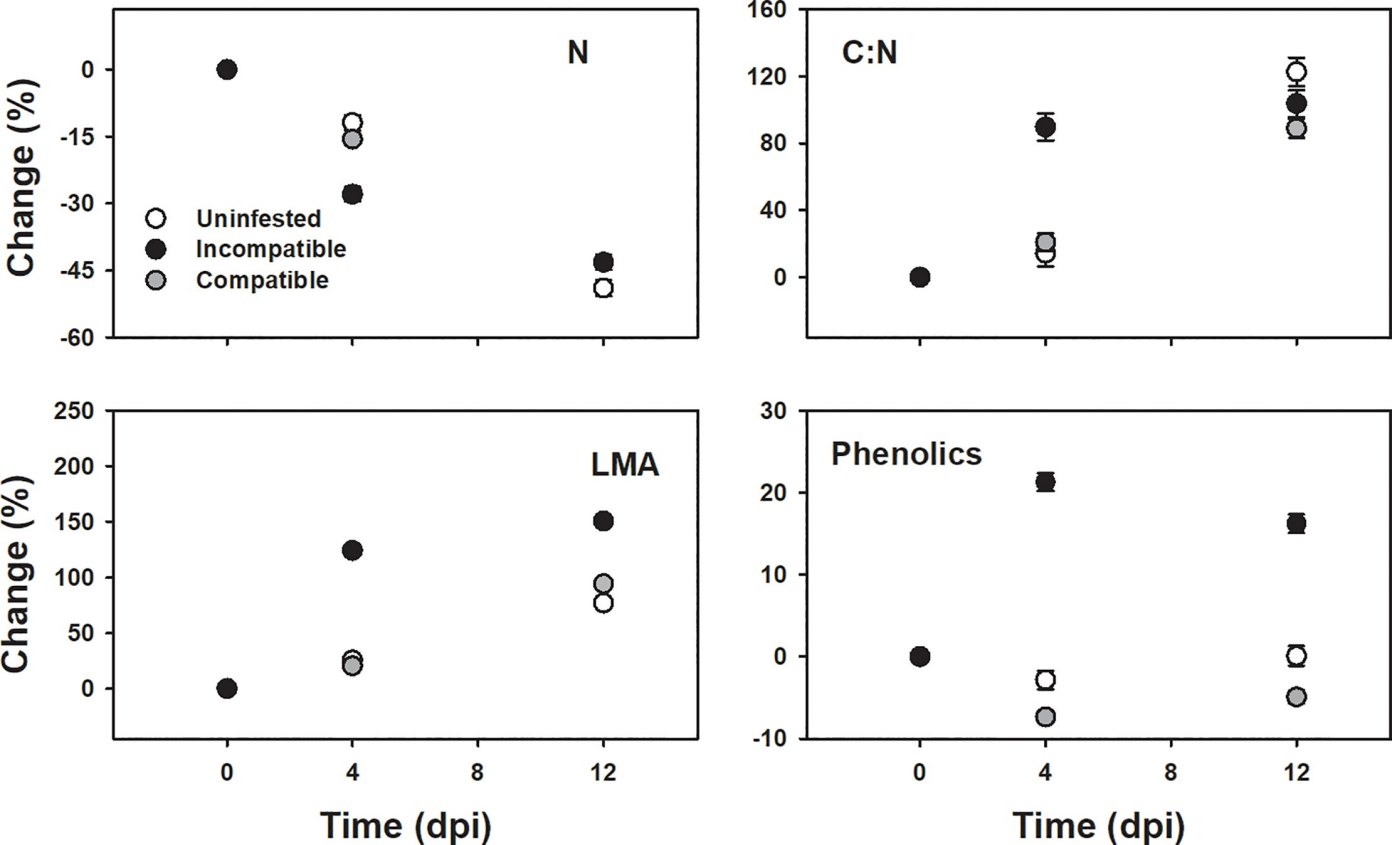
Mean ± SD of relative change, compared with the pre-infestation averages, of nitrogen (N), ratio of carbon to nitrogen (C:N), leaf mass per area (LMA) and phenolics to different infestation status (Uninfested, white circle; Incompatible, black circle; Compatible, gray circle) at zero, four and twelve days post infestation (dpi).

Infestation status and time independently and interactively affected the four plant traits we measured, as well as their relative changes to the pre-infestation values, except time alone on phenolics (Figs 3 and 4, Tables 1 and 2). Relative changes of N, C:N ratio, and LMA showed a similar trend among all treatments over time, with N decreasing and C:N and LMA increasing (Fig 3). The magnitude of the change, however, was greater 4 dpi in the incompatible interaction than the uninfested control or under compatible interactions, which were similar. Relative responses of N, C:N, and LMA across treatments became similar at 12 dpi. The relative change in phenolic concentration over time was also similar between the uninfested and compatible interaction, and the magnitude of the response was again greater in the incompatible interaction. The trends in relative phenolic content were different, however, as uninfested and compatible interactions slightly decreased, with compatible interactions decreasing more than uninfested controls, while incompatible interactions dramatically increased (Fig 4). All plant traits exhibited a similar pattern at the onset of infestation by compatible genotypes, where responses at 4 dpi were more similar to the control; plant responses to the incompatible interaction, however, were different from the control (Fig 4, Table 2). This pattern was not observed consistently at 12 dpi, except for LMA and phenolics (Fig 4, Table 2).

**Table 1:** F and *P* values of two-way ANOVA examining the effect of infestation status (Infestation status: Control, uninfested; vH-13-US and vH13-ISR [individual responses], compatible; GP, incompatible), time of infestation (Time: Four and twelve days post infestation), and their interaction on nitrogen (N), ratio of carbon to nitrogen (C:N), leaf mass per area, and phenolics.

		N (% DW)		C:N		LMA (mg cm^-2^)		Phenolics (mg GAE g^-1^)	
	df	F	*P*	F	*P*	F	*P*	F	*P*
**Infestation**	2	7.8	**<0.001**	4.5	**0.004**	469.0	**<0.001**	279.8	**<0.001**
**Time**	1	529.3	**<0.001**	180.4	**<0.001**	885.5	**<0.001**	0.9	0.901
**Infestation × Time**	2	16.0	**<0.001**	5.0	**0.002**	36.4	**<0.001**	12.1	**<0.001**

Significant values (*P* < 0.05) are bolded. df: degrees of freedom. DW, dry weight; GAE, gallic acid equivalents.

**Table 2:** F and *P* values of two-way ANOVA examining the effect of infestation status (Infestation: Control, uninfested; vH13-US and vH13-ISR [averaged response], compatible; GP, incompatible), time of post infestation (Time: Four and twelve days post infestation), and their interaction on relative changes, compared with pre-infestation averages, in nitrogen (N), ratio of carbon to nitrogen (C:N), leaf mass per area (LMA) and phenolics.

		N		C:N		LMA		Phenolics	
	df	F	*P*	F	*P*	F	*P*	F	*P*
**Infestation**	2	9.4	**<0.001**	5.4	**0.005**	705.1	**<0.001**	278.2	**<0.001**
**Time**	1	448.3	**<0.001**	167.1	**<0.001**	625.5	**<0.001**	0.01	0.901
**Infestation × Time**	2	20.8	**<0.001**	6.1	**0.002**	54.0	**<0.001**	7.1	**<0.001**

Significant values (*P* < 0.05) are bolded. df: degrees of freedom.

### Functional trait responses between virulent genotypes

**Fig 5:**
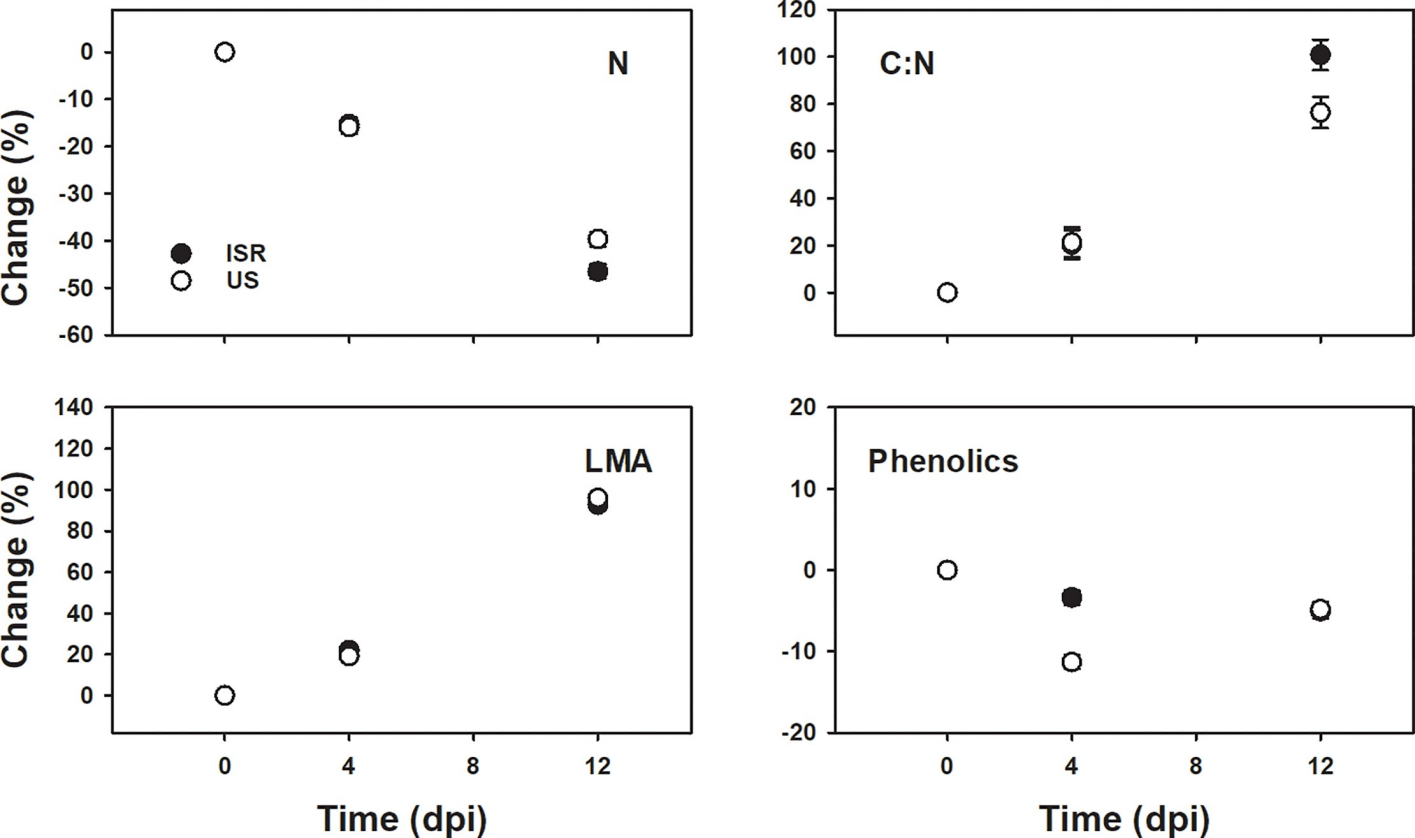
Mean ± SD of relative changes, compared with the pre-infestation averages, of nitrogen (N), ratio of carbon to nitrogen (C:N), leaf mass per area (LMA) and phenolics of compatible genotypes (H13-US, US; H13-Israel, ISR) at zero, four and twelve days post infestation (dpi).

Plant traits responded differently between compatible HF genotypes, and the response depended on the time after infestation, except for LMA (Fig 5, Table 3). At 4 dpi, plant N content and C:N levels were similar between compatible HF genotypes, but at twelve days the magnitude of change was greater for vH13-US than vH13-ISR genotypes (Fig 4). Changes in LMA were similar between HF genotypes, and the response was consistent across time (Fig 5, Table 3). The relative change in phenolics between HF genotypes was different than changes in plant N and C:N such that at 4 dpi plant phenolics were approximately three times lower in vH13-ISR population than the vH13-US population, but responses became similar 12 dpi (Fig 5).

**Table 3:** F and *P* values of two-way ANOVA examining the effect of compatible HF genotypes (Genotype: H13-US, H13-ISR), time of infestation (Time: Four and twelve days post infestation), and their interaction on relative changes, compared with pre-infestation averages, in nitrogen (N), ratio of carbon to nitrogen (C:N), leaf mass per area (LMA) and phenolics.

		N		C:N		LMA		Phenolics	
	df	F	*P*	F	*P*	F	*P*	F	*P*
**Genotype**	2	4.4	**0.036**	3.3	0.068	0.0	0.935	19.9	**<0.001**
**Time**	1	343.8	**<0.001**	111.5	**<0.001**	939.0	**<0.001**	7.9	**0.006**
**Genotype × Time**	2	6.3	**0.013**	3.9	**0.049**	1.6	0.202	22.5	**<0.001**

Significant values (*P* < 0.05) are bolded. df: degrees of freedom.

## Discussion

This investigation demonstrates that spectroscopic data can be used to effectively monitor both insect-induced gall formation and wheat-HF interactions. We tracked whole-plant functional trait responses to HF infestation and documented changes in wheat morphology and chemistry that likely influence the ability of HF to successfully induce a gall on wheat seedlings. Previous investigations have characterized the microscopic and macroscopic changes in wheat morphology, specifically near the larval feeding site, and the gene expression profiles associated with these interactions [16, 45]. Our findings provide evidence of changes in specific plant functional traits previously suspected of being associated with both incompatible and compatible wheat-HF interactions. Moreover, we found evidence of coordinated decrease in nutritional quality and increase in defenses, providing a trait-based mechanism for genetically mediated wheat resistance to HF infestation. Near infrared reflectance spectroscopy accurately predicted the relative abundance of specific groups of plant compounds previously suspected of being associated with both incompatible and compatible wheat-HF interactions [16, 45].

### Spectroscopic modelling

This study presents an approach by which specific whole plant functional trait responses to biotic stress can be monitored using reflectance spectroscopy that utilizes multiple permutations of the data, providing explicit estimates of model uncertainty. By combining high-fidelity reflectance measurements, standard morphological and biochemical analyses and robust statistical modelling, we demonstrate the potential to expand prediction capabilities of spectral data for leaf traits to detect the impact of pests on plants. Specifically, in wheat, C, N, LMA, and phenolics were very well predicted by spectral data. Wavelengths regions used in final models varied among traits (C and N, 1500–2400 nm; LMA, 400–2400 nm; phenolics, 1100–2400 nm). For N, C, and phenolics, we focused on the predominately SWIR spectral regions as previous research has shown strong relationships in these wavelength ranges with our target constituents [28, 32, 34, 46]. Utilizing this spectral region also avoids leveraging correlations among pigments and predicted variables in the visible wavelengths, instead focusing on the relationship of absorbance of organic compounds and functional groups associated with these compounds [32]. The importance of the spectral region around 1900 nm in prediction of the investigated traits is also in accordance with previous reports [28, 32, 34]. This is likely due to both the specific-absorption biochemical features contained in this spectral range and also to the high sensitivity of this region to moisture conditions of plants tissues, which are commonly directly or indirectly related to predicted leaf traits [23]. Our modelling outcomes highlight how the application and integration of approaches from multiple scientific disciplines with sensor and monitoring technologies can potentially improve the efficient of crop management and advance digital agriculture.

### Incompatible interactions

Plants use three general mechanisms to resist insect attack: 1) reduce access, 2) decrease nutritive value of the available resource, and 3) produce toxic or anti-nutritive compounds [47]. This investigation discovered evidence of all three mechanisms acting in wheat-HF incompatible interactions.

We found reduced access to the plant host via changes in leaf thickness in incompatible interactions. Incompatible interactions associated with three different *R* genes (*H6*, *H9*, *and H13*) have been shown to induce cell wall thickness in cells surrounding the larval feeding site [16]. Our results of increased LMA in plants under incompatible interactions is consistent with this finding, but also indicates that *H13*-induced cell fortification is occurring in cells beyond the feeding site.

Among the mechanisms that can potentially limit access to the resource supply are the hypersensitive response (HR) and cell wall fortification. HR causes localized cell death that isolates the parasite from nutritive tissue and has been widely observed as an induced defense response against gall-making insects [48, 49]. Although dead tissue at the feeding sites of incompatible wheat-HF interactions is difficult to detect, larval mortality and an oxidative burst on resistant plants suggests that HR may be associated with the incompatible interactions some HF *R* genes elicit [50, 51]. Although the reduced nitrogen concentrations we observed in wheat-HF incompatible interactions are consistent with the nitrogen levels associated with the HR bursts observed in other incompatible interactions [52], our investigation does not clarify whether *H13* elicits HR. If HR is part of *H13*-incompatability, it may only be a short-term resistance strategy, since 12 dpi, N levels were comparable among all treatments.

Decreasing nutritional quality of the food available to the HF also appears to play a part in *H13*-incompatabilty. Successfully infestations have resulted in pronounced increase in N containing compounds in the plant tissue near the larval feeding site three days after successful establishment [45]. At 4 dpi we found that relative changes in C:N ratios between uninfested control and compatible interactions were similar, whereas C:N ratios dramatically increased in incompatible interactions. Nitrogen, a proxy for protein, is considered the limiting nutrient for herbivorous insect growth and development [53], and given the unbalanced nitrogen stoichiometry of insects and plant content [54], an increase in the C:N ratio under incompatible interactions 4 dpi likely made it more challenging for HF to obtain the necessary quantity of nitrogen for successful growth and development, especially in the context of changes in other plant compounds.

We also found evidence that wheat upregulates production of toxins that can adversely affect the ability of an insect to survive on resistant plants. The negative effects of phenolics in general are seen in the midgut of insects [55], which seems an important target for plant resistance products. This interactions seems particularly important for the HF because the HF lacks not only a peritrophic membrane, but also of any other midgut protective structures such as the perimicrovillar membrane [56]. Therefore, microvilli are exposed to food and any toxic compounds [57] and our findings of increased phenolic compounds under incompatible interaction is consistent with the observation of midgut disruption early in the HF infestation process [56].

Depending on the *R* gene examined, more of the 80% of larvae die within four days of infestation and no larva survive after seven days [58]. An early response to the infestation would be critical to minimize the negative effects of HF infestation on plant fitness, especially considering that the negative impact of HF on wheat growth is irreversible after four days of infestation [59]. When considered together, our results indicate a coordinated response by wheat that, under incompatible interactions, limits the acquisition and quality of food during a time period critical for HR establishment and provides a mechanism for genetically mediated wheat resistance to HF infestation. Although, larvae die under incompatible interactions, the resistance genes do not prevent wheat plants from the early attack on the epidermal cell. Actually, it has been observed that the larvae try several times but are unable to establish a proper feeding site [51]. It has been proposed that this early attack could explain the growth deficit observed by resistant plants at the onset of infestation with avirulent larvae [19]. We found that responses to incompatible interactions can exceed the region of direct feeding and can be detected in all aboveground foliar area and are especially pronounced in the early infestation stages.

### Compatible interaction

Several hypotheses have been developed to explain the adaptive significance of galls. One of these is the nutrition hypothesis, which proposes that gall-inducers manipulate their host to increase nutrients and decrease the plant defensive chemicals [60]. We found that under compatible interactions HF larvae altered host plant responses, and that successfully galled plants behaved similarly to uninfested plants, especially at the beginning of the infestation. Thus, gall inducers likely inhibit the normal physiological responses of the plant during incompatible interactions to exploit the resources they need for successful development.

The phenotypic changes we observed on galled Molly plants were dependent, in part, on the HF genotype. At 4 dpi plants from different genotypes of compatible interactions differed only in phenolic content, with a lower phenolic content in plants infested with vH13-ISR than vH13-US. Lower phenolic concentrations may be associated with the higher infestation success of vH13-ISR compared to vH13-US genotype on H13-plants observed in preliminary studies (VCM, personal observation). However, we found no statistically significant differences in larval performance, measured as body length, among the different HF genotypes at either 4- or 12-dpi (genotype effect: *t =* 0.08, *p =* 0.93; genotype x dpi effect: *t* = -1.28, *p* = 0.23). These findings suggest that phenolic compounds may play a role in HF establishment, and establishment success may vary among virulent genotypes, but once established, HF genotypes preform similarly. Examining changes in specific phenolics in wheat in response to virulent and avirulent infestations and HF midgut antioxidant capacity may provide a more mechanistic physiological explanation for the mechanisms underlying HF establishment success on susceptible wheat varieties.

In conclusion, our results suggest that reflectance spectroscopy is able to detect and quantify changes in wheat physiological responses to virulent and avirulent HF populations. We found that coordinated changes in plant nutritional quality and defenses are likely critical factors determining the capability of wheat to resist HF infestation. These findings help shed light on factors that influence genetically mediated host plant resistance. While at least 7% of all genes in the HF genome code for effector proteins and several candidate effector genes have been identified [14, 61, 62] the function of the majority of these effector proteins is unknown. Nevertheless, bio-assays that express these proteins inside the plant have been designed [63] and we suggest that reflectance spectroscopy in combination with these techniques could be a powerful tool to study wheat functional trait responses to HF infestation and to determine whether *R* gene resistance strategies are convergent or divergent. Ultimately, incorporating spectroscopy into studies of plant-herbivore interactions has the potential to provide a better understanding of trait-based resistance mechanisms of plants to galling herbivores.

## Supporting information

S1 Table Summary statistics (Mean, standard deviation, minimum, and maximum) for functional traits used in the chemometric modelling. LMA, leaf mass per area; GA, gallic acid equivalents; SD, standard deviation).
(PDF)

## References

[1] Basu S, Varsani S, Lewis J. Altering plant defenses: Herbivore-associated molecular patterns and effector arsenal of chewing herbivores. MPMI 2017; 31:13–21. 10.1094/MPMI-07-17-0183-FI 28840787

[2] Tooker JF, Rohr JR, Abrahamson WG, De Moraes CM. Gall insects can avoid and alter indirect plant defenses. New Phytol. 2008; 178:657–671. 10.1111/j.1469-8137.2008.02392.x 18331430

[3] Stone GN, Schönrogge K. The adaptive significance of insect gall morphology. Trends Ecol Evol. 2003; 18:512–522.

[4] Hartley SE. The chemical composition of plant galls: are levels of nutrients and secondary compounds controlled by the gall-former? Oecologia. 2006; 113:492–501. 10.1007/s004420050401 28308028

[5] Tooker JF, De Moraes CM. Feeding by Hessian fly Mayetiola destructor (Say) larvae does not induce plant indirect defences. Ecol Entomol. 2007; 32:153–161.

[6] Giron D, Huguet E, Stone GN, Body M. Insect-induced effects on plants and possible effectors used by galling and leaf-mining insects to manipulate their host-plant. J Insect Physiol. 2016; 84:70–89. 10.1016/j.jinsphys.2015.12.009 26723843

[7] Oates CN, Denby KJ, Myburg AA, Slippers B, Naidoo S. Insect gallers and their plant hosts: from omics data to systems biology. Int J Mol Sci. 2016; 17:1891 10.3390/ijms17111891 27869732 PMC5133890

[8] Berzonsky WA, Ding H, Haley SD, Harris MO, Lamb RJ, McKenzie RIH, et al Breeding wheat for resistance to insects In: Janick J, editor. Plant Breeding Reviews, volume 22 New York: John Wiley & Sons; 2003 pp. 221–296.

[9] Cambron SE, Buntin GD, Weisz R, Holland JD, Flanders KL, Schemerhorn BJ, et al Virulence in hessian fly (Diptera: Cecidomyiidae) field collections from the Southeastern United States to 21 resistance genes in wheat. J Econ Entomol. 2010; 103:2229–2235. 10.1603/ec10219 21309248

[10] Stuart JJ, Chen MS, Shukle R, Harris MO. Gall midges (Hessian flies) as plant pathogens. Annu Rev Phytopathol. 2012; 50:339–357. 10.1146/annurev-phyto-072910-095255 22656645

[11] Stuart JJ. Insect effectors and gene-for-gene interactions with host plants. Curr Opin in Insect Sci. 2015; 9:56–61. 10.1016/j.cois.2015.02.010 32846709

[12] Liu XM, Gill BS, Chen M-S. Hessian fly resistance gene H13 is mapped to a distal cluster of resistance genes in chromosome 6DS of wheat. Theor Appl Genet. 2005; 111:243–249. 10.1007/s00122-005-2009-5 15942758

[13] Patterson F, Maas F III, Foster JE, Ratcliffe R, Cambron S, Safranski G, et al Registration of eight Hessian fly resistant common winter wheat germplasm lines (Carol, Erin, Flynn, Iris, Joy, Karen, Lola, and Molly). Crop Sci. 1994; 34:315–316.

[14] Aggarwal R, Subramanyam S, Zhao CY, Chen MS, Harris MO, Stuart JJ. Avirulence effector discovery in a plant galling and plant parasitic arthropod, the Hessian fly (*Mayetiola destructor)*. Plos One. 2014; 9:e100958 10.1371/journal.pone.0100958 24964065 PMC4071006

[15] Harris MO, Freeman TP, Rohfritsch O, Anderson KG, Payne SA, Moore JA. Virulent Hessian fly (Diptera: Cecidomyiidae) larvae induce a nutritive tissue during compatible interactions with wheat. Ann Entomol Soc Am. 2006; 99:305–316.

[16] Harris MO, Freeman TP, Moore JA, Anderson KG, Payne SA, Anderson KM, et al H-gene-mediated resistance to Hessian fly exhibits features of penetration resistance to fungi. Phytopathology. 2010; 100:279–289. 10.1094/PHYTO-100-3-0279 20128702

[17] Williams CE, Nemacheck JA, Shukle JT, Subramanyam S, Saltzmann KD, Shukle RH. Induced epidermal permeability modulates resistance and susceptibility of wheat seedlings to herbivory by Hessian fly larvae. J Exp Bot. 2011; 62:4521–4531. 10.1093/jxb/err160 21659664 PMC3170548

[18] Liu X, Bai J, Huang L, Zhu L, Liu X, Weng N, et al Gene expression of different wheat genotypes during attack by virulent and avirulent Hessian fly (*Mayetiola destructor*) larvae. J Chem Ecol. 2007; 33:2171–2194. 10.1007/s10886-007-9382-2 18058177

[19] Bronner N. The role of nutritive cells in the nutrition of cynipids and cecidomyiids In: Shorthouse JD, Rohfritsch O, editors. Biology of insect-induced galls. New York: Oxford University Press; 1992 pp. 118–140.

[20] Anderson K, Harris M. Does R gene resistance allow wheat to prevent plant growth effects associated with Hessian fly (Diptera: Cecidomyiidae) attack? J Econ Entomol. 2006; 99:1842–1853. 10.1603/0022-0493-99.5.1842 17066821

[21] Leszczynski B, Tjallingii W, Dixon A, Swiderski R. Effect of methoxyphenols on grain aphid feeding behaviour. Entomol Exp Appl. 1995; 76:157–162.

[22] Sankaran S, Mishra A, Ehsani R, Davis C. A review of advanced techniques for detecting plant diseases. Comput Electron Ag. 2010; 72:1–13.

[23] Cotrozzi L, Townsend PA, Pellegrini E, Nali C, Couture JJ. Reflectance spectroscopy: a novel approach to better understand and monitor the impact of air pollution on Mediterranean plants. Environ Sci Pollut Res. 2018; 25:8249–8267. 10.1007/s11356-017-9568-2 28699011

[24] Couture JJ, Serbin SP, Townsend PA. Spectroscopic sensitivity of real‐time, rapidly induced phytochemical change in response to damage. New Phytol. 2013; 198:311–319. 10.1111/nph.12159 23384059

[25] Arens N, Backhaus A, Döll S, Fischer S, Seiffert U, Mock H-P. Non-invasive presymptomatic detection of Cercospora beticola infection and identification of early metabolic responses in sugar beet. Front Plant Sci. 2016; 7:1377 10.3389/fpls.2016.01377 27713750 PMC5031787

[26] Cotrozzi L, Couture JJ, Cavender-Bares J, Kingdon CC, Fallon B, Pilz G, et al Using foliar spectral properties to assess the effects of drought on plant water potential. Tree Physiol. 2017; 37:1582–1591. 10.1093/treephys/tpx106 29036552

[27] Couture JJ, Singh A, Charkowski AO, Groves RL, Gray S, Bethke PC, et al Integrating spectroscopy with potato disease management. Plant Dis. 2018; 102:2233–2240. 10.1094/PDIS-01-18-0054-RE 30145947

[28] Curran PJ. Remote sensing of foliar chemistry. Remote Sens Environ. 1989; 30:271–278.

[29] Peñuelas J, Filella I. Visible and near-infrared reflectance techniques for diagnosing plant physiological status. Trends Plant Sci. 1998; 3:151–156.

[30] Asner GP, Martin RE. Spectral and chemical analysis of tropical forests: Scaling from leaf to canopy levels. Remote Sens Environ. 2008; 112:3958–3970.

[31] Serbin SP, Dillaway DN, Kruger EL, Townsend PA. Leaf optical properties reflect variation in photosynthetic metabolism and its sensitivity to temperature. J Exp Bot. 2012; 63:489–502. 10.1093/jxb/err294 21984647 PMC3245480

[32] Couture JJ, Singh A, Rubert‐Nason KF, Serbin SP, Lindroth RL, Townsend PA. Spectroscopic determination of ecologically relevant plant secondary metabolites. Methods Ecol Evol. 2016; 7:1402–1412.

[33] Foley WJ, McIlwee A, Lawler I, Aragones L, Woolnough AP, Berding N. Ecological applications of near infrared reflectance spectroscopy–a tool for rapid, cost-effective prediction of the composition of plant and animal tissues and aspects of animal performance. Oecologia. 1998; 116:293–305. 10.1007/s004420050591 28308060

[34] Serbin SP, Singh A, McNeil BE, Kingdon CC, Townsend PA. Spectroscopic determination of leaf morphological and biochemical traits for northern temperate and boreal tree species. Ecol Appl. 2014; 24:1651–1669. 10.1890/13-2110.1 29210229

[35] Ainsworth EA, Gillespie KM. Estimation of total phenolic content and other oxidation substrates in plant tissues using Folin–Ciocalteu reagent. Nat Protoc. 2007; 2:875–877. 10.1038/nprot.2007.102 17446889

[36] Wold S, Ruhe A, Wold H, Dunn WJ III. The collinearity problem in linear regression. The partial least squares (PLS) approach to generalized inverses. SIAM J Sci and Stat Comput. 1984; 5:735–743.

[37] Wold S, Sjöström M, Eriksson L. PLS-regression: a basic tool of chemometrics. Chemometr Intell Lab Syst. 2001; 58:109–130.

[38] Grossman Y, Ustin S, Jacquemoud S, Sanderson E, Schmuck G, Verdebout J. Critique of stepwise multiple linear regression for the extraction of leaf biochemistry information from leaf reflectance data. Remote Sens Environ. 1996; 56:182–193.

[39] Bolster KL, Martin ME, Aber JD. Determination of carbon fraction and nitrogen concentration in tree foliage by near infrared reflectances: A comparison of statistical methods. Can J For Res. 1996; 26:590–600.

[40] Atzberger C, Guérif M, Baret F, Werner W. Comparative analysis of three chemometric techniques for the spectroradiometric assessment of canopy chlorophyll content in winter wheat. Comput Electron Agr. 2010; 73:165–173.

[41] Asner GP, Martin RE, Tupayachi R, Emerson R, Martinez P, Sinca F, et al Taxonomy and remote sensing of leaf mass per area (LMA) in humid tropical forests. Ecol Appl. 2011; 21:85–98. 21516890 10.1890/09-1999.1

[42] Chen S, Hong X, Harris CJ, Sharkey PM. Spare modeling using orthogonal forest regression with PRESS statistic and regularization. IEEE Trans Syst Man Cybern. 2004; 34:898–911. 10.1109/tsmcb.2003.817107 15376838

[43] Chong I-G, Jun C-H. Performance of some variable selection methods when multicollinearity is present. Chemometr Intell Lab Syst. 2005; 78:103–112.

[44] Mevik BH, Wehrens R. The pls package: Principal component and partial least squares regression in R. J Stat Soft. 2007; 18:1–23.

[45] Zhu L, Liu XM, Liu X, Jeannotte R, Reese JC, Harris M, et al Hessian fly (*Mayetiola destructor*) attack causes a dramatic shift in carbon and nitrogen metabolism in wheat. Mol Plant Microbe Interact. 2008; 21:70–78. 10.1094/MPMI-21-1-0070 18052884

[46] Silvia-Perez V, Molero G, Serbin SP, Condon AG, Renyolds MP, Furbank RT, et al Hyperspectral reflectance as a tool to measure biochemical and physiological traits in wheat. J Exp Bot. 2018; 69:483–496 10.1093/jxb/erx421 29309611 PMC5853784

[47] Chen M-S. Inducible direct plant defense against insect herbivores: a review. Insect Sci. 2008; 15:101–114. 10.1111/1744-7917.12436 28035791

[48] Fernandes GW. Hypersensitivity—a neglected plant-resistance mechanism against insect herbivores. Environ Entomol. 1990; 19:1173–1182.

[49] Höglund S. Timing of growth determines fitness and performance of a galling insect on willow. Ecol Entomol. 2014; 39:159–167.

[50] Shukle RH, Grover PB, Mocelin G. Responses of susceptible and resistant wheat associated with Hessian fly (Diptera, Cecidomyiidae) infestation. Environ Entomol. 1992; 21:845–853.

[51] Grover PB Jr. Hypersensitive response of wheat to the Hessian fly. Entomol Exp Appl. 1995 74:283–294.

[52] Santos JC, Alves-Silva E, Cornelissen TG, Fernandes GW. Differences in leaf nutrients and developmental instability in relation to induced resistance to a gall midge. Arthropod Plant Interact. 2017; 11:163–170.

[53] Mattson W. Herbivory in relation to plant nitrogen content. Annu Rev Ecol Syst. 1980; 11:119–161.

[54] Fagan WF, Siemann E, Mitter C, Denno RF, Huberty AF, Woods HA, Elser JJ. Nitrogen in insects: implications for trophic complexity and species diversification. Am Nat. 2002; 160:784–802. 10.1086/343879 18707465

[55] Simmonds MS. Flavonoid–insect interactions: recent advances in our knowledge. Phytochemistry. 2003; 64:21–30. 12946403 10.1016/s0031-9422(03)00293-0

[56] Shukle RH, Subramanyam S, Saltzmann KA, Williams CE. Ultrastructural changes in the midguts of Hessian fly larvae feeding on resistant wheat. J Insect Physiol. 2010; 56:754–760. 10.1016/j.jinsphys.2010.01.005 20116382

[57] Nation JL. Insect Physiology and Biochemistry Boca Raton: CRC Press; 2008.

[58] Chen M-S, Liu X, Wang H, El-Bouhssini M. Hessian fly (Diptera: Cecidomyiidae) interactions with barley, rice, and wheat seedlings. J Econ Entomol. 2009; 102:1663–1672. 10.1603/029.102.0434 19736782

[59] Byers RA, Gallun RL. Ability of Hessian fly Diptera:Cecidomyiidae to stunt winter-wheat .1. Effect of larval feeding on elongation of leaves. J Econ Entomol. 1972; 65:955–958.

[60] Price PW, Fernandes GW, Waring GL. Adaptive nature of insect galls. Environ Entomol. 1987; 16:15–24.

[61] Zhao C, Escalante LN, Chen H, Benatti TR, Qu J, Chellapilla S, et al A massive expansion of effector genes underlies gall-formation in the wheat pest *Mayetiola destructor*. Curr Biol. 2015; 25:613–620. 10.1016/j.cub.2014.12.057 25660540

[62] Zhao C, Shukle R, Navarro-Escalante L, Chen M, Richards S, Stuart JJ. Avirulence gene mapping in the Hessian fly (*Mayetiola destructor*) reveals a protein phosphatase 2C effector gene family. J Insect Physiol. 2016; 84:22–31. 10.1016/j.jinsphys.2015.10.001 26439791

[63] Navarro L. Discovery and functional analyses of Hessian fly effector-encoding genes. Doctoral dissertation, Purdue University. 2016. Available from: https://docs.lib.purdue.edu/open_access_dissertations/1393/

