# Supplementary material for: Spectral characterization of wheat functional trait responses to Hessian fly: Mechanisms for trait-based resistance
Source: PLoS One. 2019 Aug 22;14(8):e0219431. doi: 10.1371/journal.pone.0219431 (PMC6705800; doi:10.1371/journal.pone.0219431)
Supplement: S1 Table — LMA, leaf mass per area; GA, gallic acid equivalents; SD, standard deviation). (PDF) [file pone.0219431.s001.pdf]

Supporting Information Table 1. Summary statistics (Mean, standard deviation, minimum, and maximum) for functional traits used in the chemometric modelling. LMA, leaf mass per area; GA, gallic acid equivalents; SD, standard deviation).

**Functional trait**	**Mean**	**SD**	**Minimum**	**Maximum**
**Nitrogen (% dw)**	4.29	1.09	1.91	6.15
**Carbon (% dw)**	40.15	1.04	37.89	42.96
**LMA (mg cm^{-2})**	4.88	1.59	2.51	8.41
**Phenols (mg GAE g^{-1})**	8.81	0.97	7.1	11.07
